# Supplementary material for: A pharmacist-led interprofessional medication adherence program improved adherence to oral anticancer therapies: The OpTAT randomized controlled trial
Source: PLoS One. 2024 Jun 7;19(6):e0304573. doi: 10.1371/journal.pone.0304573 (PMC11161104; doi:10.1371/journal.pone.0304573)
Supplement: S1 Appendix — (DOCX) [file pone.0304573.s001.docx]

**Appendix 1:** Design of the adherence part of the OpTAT study adapted from Bandiera et al.[1]


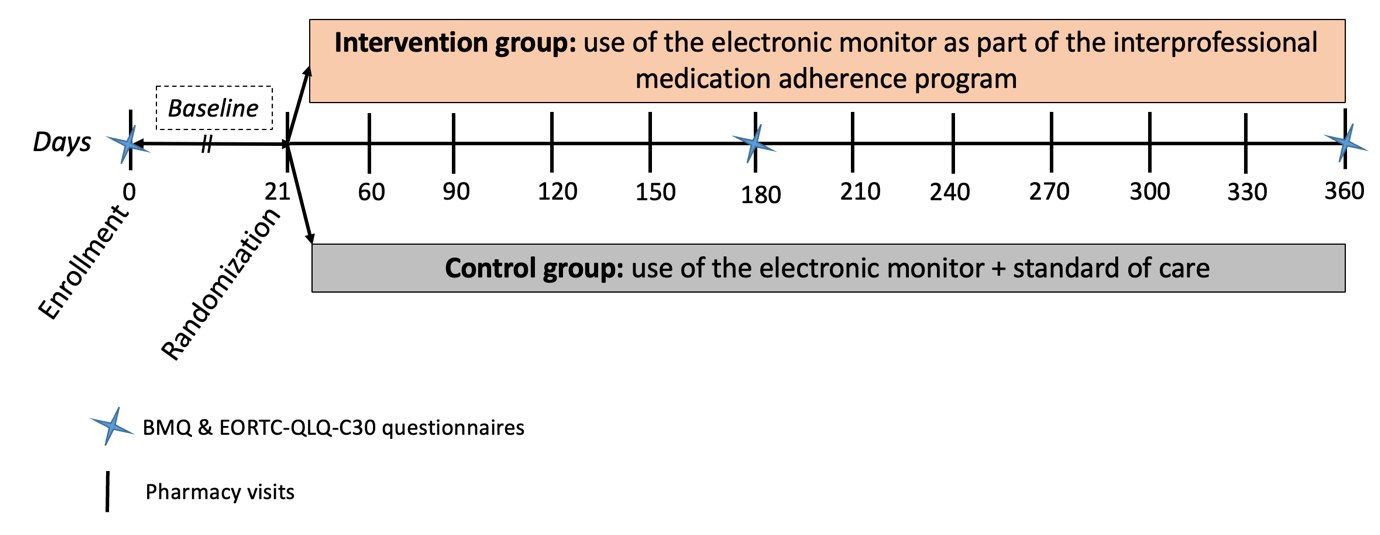


NB: BMQ= Beliefs about Medicines Questionnaire; EORTC-QLQ-C30= European Organization for Research and Treatment of Cancer Quality of Life Questionnaire, version 3.0

1. Bandiera, C., et al., *Optimizing Oral Targeted Anticancer Therapies Study for Patients With Solid Cancer: Protocol for a Randomized Controlled Medication Adherence Program Along With Systematic Collection and Modeling of Pharmacokinetic and Pharmacodynamic Data.* JMIR Res Protoc, 2021. **10**(6): p. e30090.
