# Supplementary figures and images for: A pharmacist-led interprofessional medication adherence program improved adherence to oral anticancer therapies: The OpTAT randomized controlled trial
Source: PLoS One. 2024 Jun 7;19(6):e0304573. doi: 10.1371/journal.pone.0304573 (PMC11161104; doi:10.1371/journal.pone.0304573)

**Appendix 2:** main reasons for non-participation reported by 103/111 patients who refused to participate


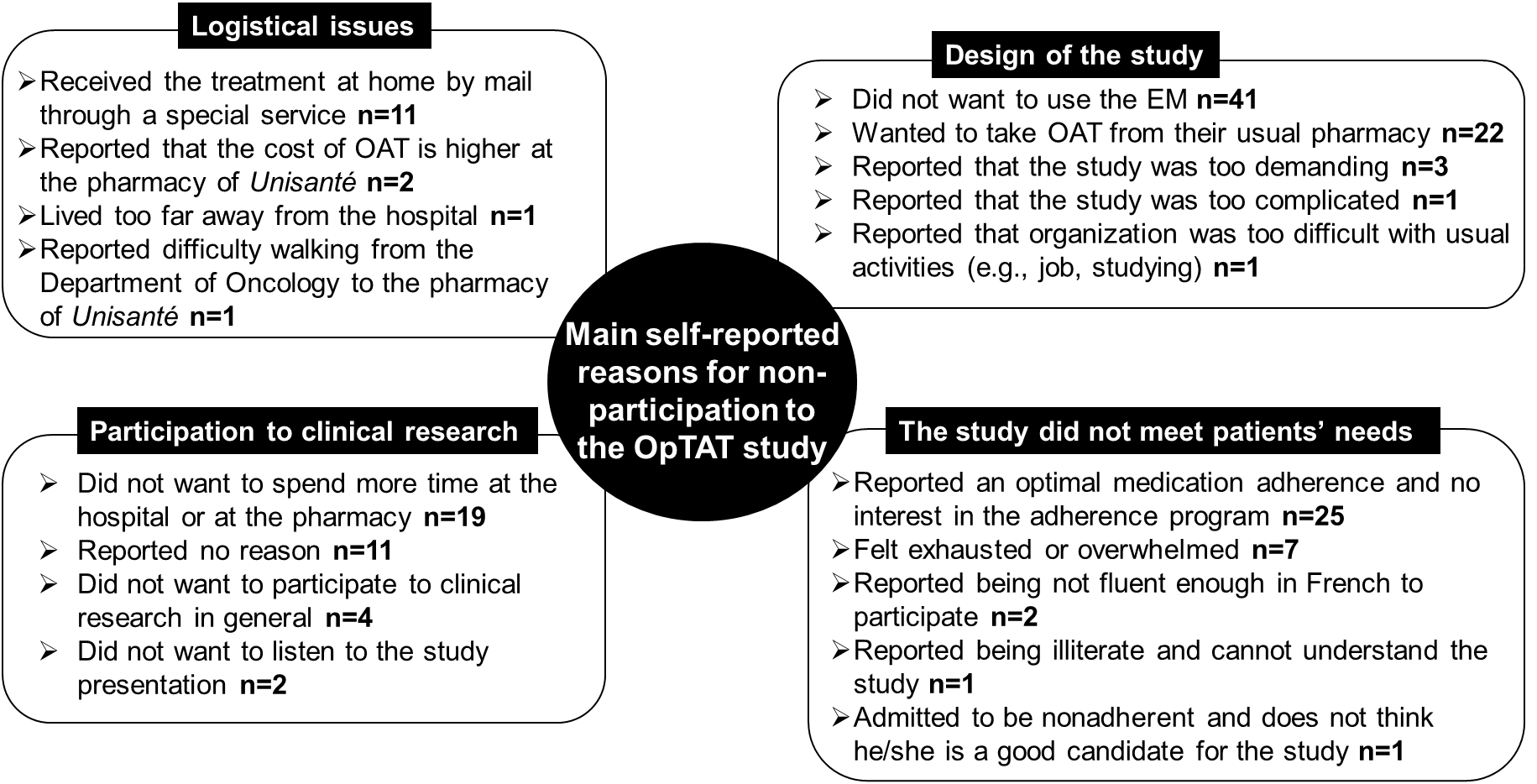

Supplement: S2 Appendix — (DOCX) [file pone.0304573.s002.docx]
