# Supplementary material for: A pharmacist-led interprofessional medication adherence program improved adherence to oral anticancer therapies: The OpTAT randomized controlled trial
Source: PLoS One. 2024 Jun 7;19(6):e0304573. doi: 10.1371/journal.pone.0304573 (PMC11161104; doi:10.1371/journal.pone.0304573)

**Appendix 3:** PKI persistence and adherence in both groups since randomization.

Kaplan Meier curves showing PKI persistence (censoring times are represented with dots on the dotted lines) and GEE model showing PKI adherence in the intervention group (red line) and control group (blue line) since randomization.
NB: the red and blue lines on the background show empirical adherence in the intervention and control groups respectively.


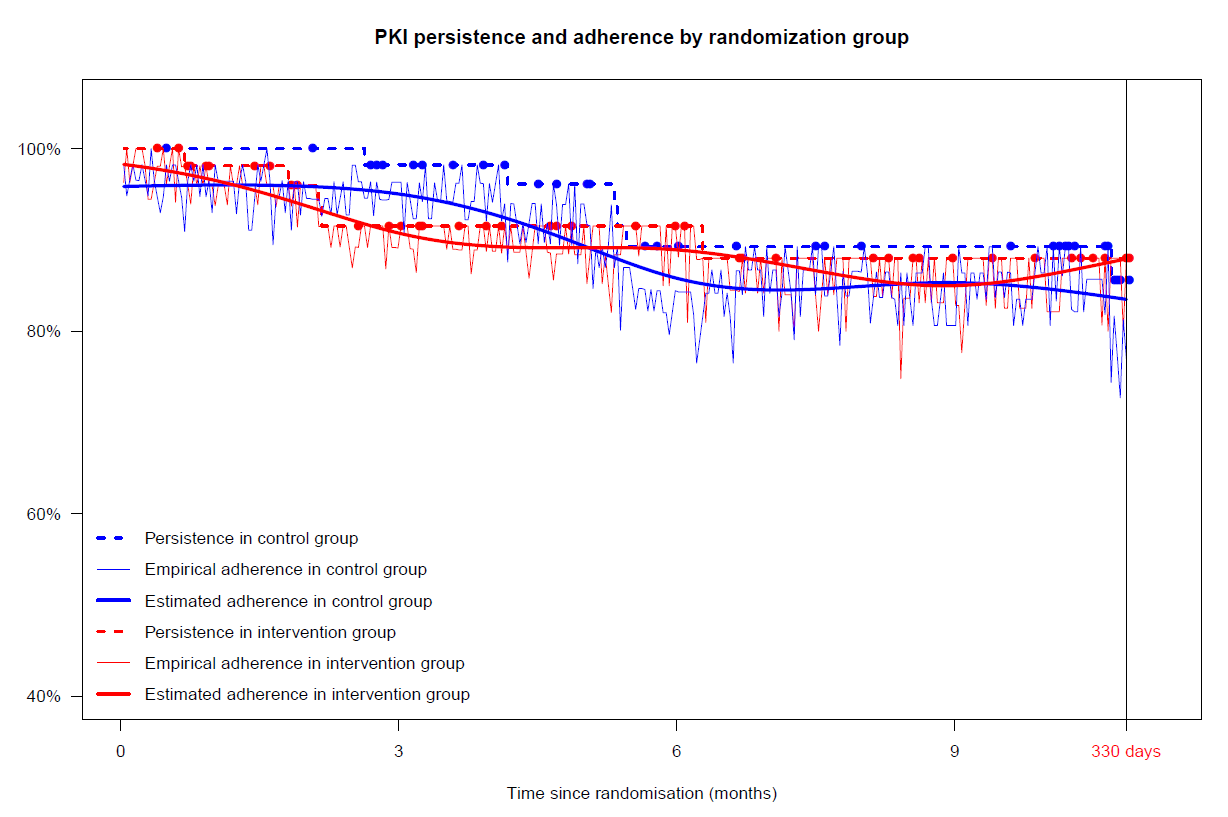

Supplement: S3 Appendix — (DOCX) [file pone.0304573.s003.docx]
