# Supplementary material for: A pharmacist-led interprofessional medication adherence program improved adherence to oral anticancer therapies: The OpTAT randomized controlled trial
Source: PLoS One. 2024 Jun 7;19(6):e0304573. doi: 10.1371/journal.pone.0304573 (PMC11161104; doi:10.1371/journal.pone.0304573)
Supplement: S4 Appendix — (DOCX) [file pone.0304573.s004.docx]

**Appendix 4:** Questionnaire scores in patients included in the intervention versus control groups at 6- and 12-month post-inclusion

NB: The BMQ is composed of 18 questions rated on a 5-point Likert scale from 1 “Strongly agree” to 5 “Strongly disagree”. For each scale, a total score was calculated by adding the reverse scores of the questions, which ranged from 5 to 25 for perceived necessity and concerns, and from 4 to 20 for perceived over-prescribing and prejudices[1]. Higher scores indicate stronger beliefs[1].

The EORTC-QLQ-C30 questionnaire is composed of 30 questions, rated on a 4-point Likert scale from 1 “Not at all” to 4 “Very much”; and perceived global health status (2 questions) rated on a 7-point Likert scale from 1 “Very poor” to 7 “Excellent”. Each scale was scored as follows: the mean of the score per question (RawScore, RS) was linearly transformed to a 0-100 score (S) as defined by the guidelines (i.e., for the functional scale, S=(1-(RS-1)/3)x100; for the symptom scale, S=((RS-1)/3)x100 and for the global health status, S=((RS-1)/6)x100)[2, 3]. A high score for the functional scale indicates a healthy level of functioning, a high score for the global health status indicates high QoL and a high score in the symptom scale indicates a high level of symptomatology[3].

1. BMQ and EORTC-QlQ-C30 scores at 6-month post-inclusion in both groups

| Variables, at 6-month post-inclusion | Control  (n=60, 50.8%) | Intervention  (n=58, 49.2%) | p-value  (Welch’s t-test) |
| --- | --- | --- | --- |
| BMQ_necessity_6m | 10.1 (±3) | 9.7 (±4.3) | 0.758 |
| missing values | 45 | 45 |  |
| BMQ_concerns_6m | 14.9 (±4.1) | 15.6 (±4.6) | 0.666 |
| missing values | 45 | 44 |  |
| BMQ_prejudices_6m | 15.5 (±3.2) | 15.3 (±2.5) | 0.819 |
| missing values | 45 | 47 |  |
| BMQ_overprescribing_6m | 14.3 (±2.7) | 14.3 (±2.3) | 0.979 |
| missing values | 45 | 45 |  |
| EORTC_funtioning_6m | 81.7 (±13.7) | 83.9 (±12.4) | 0.677 |
| missing values | 46 | 46 |  |
| EORTC_symptoms_6m | 24.1 (±14) | 24.1 (±22.1) | 0.995 |
| missing values | 45 | 46 |  |
| EORTC_global_health_6m | 66.7 (±17.5) | 60.9 (±20.2) | 0.432 |
| missing values | 45 | 45 |  |

1. BMQ and EORTC-QlQ-C30 scores at 12-month post-inclusion in both groups

| Variables, at 12-month post-inclusion | Control  (n=60, 50.8%) | Intervention  (n=58, 49.2%) | p-value  (Welch’s t-test) |
| --- | --- | --- | --- |
| BMQ_necessity_12m | 9.7 (±3.5) | 9.9 (±4) | 0.791 |
| missing values | 19 | 24 |  |
| BMQ_concerns_12m | 14.3 (±4.6) | 15.8 (±4.8) | 0.174 |
| missing values | 22 | 26 |  |
| BMQ_prejudices_12m | 14 (±3) | 14.1 (±3.1) | 0.962 |
| missing values | 20 | 24 |  |
| BMQ_overprescribing_12m | 14.2 (±2.9) | 14.1 (±3) | 0.839 |
| missing values | 20 | 24 |  |
| EORTC_functioning_12m | 74.8 (±16.9) | 73.7 (±18.9) | 0.822 |
| missing values | 33 | 36 |  |
| EORTC_symptoms_12m | 25.3 (±16.6) | 28.4 (±19.9) | 0.563 |
| missing values | 35 | 35 |  |
| EORTC_global_healt_12m | 67.6 (±16.7) | 56.7 (±23.2) | 0.060 |
| missing values | 33 | 33 |  |

**Appendix 4:** Questionnaire scores between patients who refused (at the enrolment date) and those who accepted to participate (at the study inclusion) in the OpTAT study

| Variables at the enrollment date or at study inclusion | Patients who refused  (n=103, 46.6%) | Patients who accepted  (n=118, 53.4%) | p-value  (Welch’s t-test) |
| --- | --- | --- | --- |
| BMQ_necessity_0m | 10.1 (±3.4) | 9.2 (±3.6) | 0.162 |
| missing values | 62 | 19 |  |
| BMQ_concerns_0m | 15.1 (±5) | 15.2 (±4.6) | 0.890 |
| missing values | 67 | 15 |  |
| BMQ_prejudices_0m | 14.5 (±2.8) | 14.7 (±2.7) | 0.658 |
| missing values | 62 | 18 |  |
| BMQ_overprescribing_0m | 14.1 (±3.2) | 13.8 (±3.1) | 0.696 |
| missing values | 63 | 14 |  |
| EORTC_functioning_0m | 75.6 (±16.2) | 79.7 (±15.4) | 0.271 |
| missing values | 74 | 59 |  |
| EORTC_symptoms_0m | 26.8 (±13.4) | 24.9 (±17.1) | 0.580 |
| missing values | 74 | 61 |  |
| EORTC_global_health_0m | 59.9 (±18.4) | 66.1 (±20.2) | 0.143 |
| missing values | 72 | 55 |  |

References

1. Fall, E., et al., *Validation of the French version of the Beliefs about Medicines Questionnaire (BMQ) among diabetes and HIV patients.* 2014. **64**(6): p. 335 - 343.

2. Aaronson, N.K., et al., *The European Organization for Research and Treatment of Cancer QLQ-C30: a quality-of-life instrument for use in international clinical trials in oncology.* J Natl Cancer Inst, 1993. **85**(5): p. 365-76.

3. Fayers PM, A.N., Bjordal K, Groenvold M, Curran D, Bottomley A, on and b.o.t.E.Q.o.L. Group., *The EORTC QLQ-C30 Scoring Manual (3rd Edition)*. 2001: European Organisation for Research and Treatment of Cancer, Brussels.
