# Supplementary material for: A pharmacist-led interprofessional medication adherence program improved adherence to oral anticancer therapies: The OpTAT randomized controlled trial
Source: PLoS One. 2024 Jun 7;19(6):e0304573. doi: 10.1371/journal.pone.0304573 (PMC11161104; doi:10.1371/journal.pone.0304573)
Supplement: Attachment — Submitted filename: Point-by-point responses to reviewersOpTAT_02.01.2024.pdf [file pone.0304573.s005.pdf]

UNIVERSITÉ DE GENÈVE
FACULTÉ DES SCIENCES
Section des sciences pharmaceutiques

ISPSO
INSTITUT DES SCIENCES PHARMACEUTIQUES DE SUISSE OCCIDENTALE

Marie Paule Schneider
Professeure associée

Phone: 022 379 53 16
Marie.Schenider@unige.ch

Editorial Board
PLOS ONE

January 2nd, 2024

Dear editor and reviewers,
We thank you for your comments on our manuscript entitled "**A pharmacist-led interprofessional medication adherence program improved adherence to oral anticancer therapies: The OpTAT randomized controlled trial**". We are pleased to submit 1) a rebuttal letter that responds to each point raised by the academic editor and reviewers, 2) a marked-up copy of the manuscript that highlights changes made to the original version, 3) an unmarked version of the revised paper without tracked changes.

<u>Reviewers' comments</u>
Reviewer #1:

**Reviewer's comment**	**Authors' response**
Introduction<br>1. Why is it necessary to investigate the adherence of protein kinase inhibitors (PKIs) in the context of solid tumors when the current body of literature has focused on their adherence in chronic myeloid leukemia (CML)?	Since the launched of imatinib on the market in 2001, an increasing number of oral anticancer therapies (OATs) have been marketed for a large variety of solid cancers. While many studies have monitored adherence to protein kinase inhibitors (PKIs) in chronic myeloid leukemia (CML), there is a gap in the literature regarding adherence to the other PKIs prescribed for solid tumors. Data on adherence to palliative treatment lines is lacking in solid cancers.<br>It is thus necessary to investigate adherence to PKIs in solid cancers to bridge this gap. This statement was indicated in the introduction lines 105-108. We added a comment regarding the gap in the literature lines 107-108.
What justifies the need for a distinct examination of PKI adherence in solid tumors and why can't the findings from CML studies be directly extrapolated?	There is a need to better understand adherence to PKIs in solid tumors as: 1) the diversity of PKIs prescribed is much larger in solid cancers than in CML, for which mainly imatinib, dasatinib, nilotinib, ponatinib and bosutinib are prescribed, 2)

	in CML, the PKIs treatment objectives are primarily neo-adjuvant or adjuvant. In solid tumors, PKIs are also prescribed as palliative treatments. The type of diagnostic, the objective of the treatment, the length of the treatment (short versus long term) belong to the 700 determinants of medication adherence[1]. Thus, it is necessary to investigate adherence to PKIs in other diseases than CML; 3) in the scientific literature, time of follow-up is often short and sample sizes are small, the methodology for measuring medication adherence varies (e.g., pill-count, pharmacy refill, electronic monitoring, self-report questionnaires), yet patient self-report is being used most. Moreover, adherence measured with self-report questionnaires is often higher than estimated with objective methods [2, 3]. Ultimately, operational definitions to calculate medication adherence outcomes across the three phases (i.e., initiation, implementation and persistence) are often lacking, especially regarding the distinction between PKIs implementation and persistence[4-6].<br><br>For these reasons, the findings in studies reporting medication adherence to PKIs in CML cannot be extrapolated in solid cancer patients.
Furthermore, what insights have previous studies provided regarding PKI adherence in CML?<br><br>Incorporating these aspects into the introduction would help elucidate the significance of this study.	In 2016, Greer et al. estimated in their systematic review that adherence to OATs (mainly endocrine therapies to treat breast cancer and PKIs for CML) varies from 46% to 100%[4]. In their systematic review, Huang et al. reported that adherence to OATs –mainly PKIs for CML– could be as poor as 23%[5]. Both reviews were cited in the introduction, lines 97-98. The limitations of the published studies in the field of adherence to PKIs have been described in the previous answer of this letter.<br>The significance of this study is explained in the introduction, and through the gap in

	the literature as explained on lines 105-108.
Methods<br>1. Could you explain your process for identifying and reaching out to potential pharmacists to obtain informed consent?	The pharmacists did not have to provide a specific consent as they delivered the intervention as part of their usual practice at the community pharmacy.<br>The process for recruiting patients is detailed in the published protocol, cited line 164[7]: "Oncologists were asked to refer eligible patients to a pharmacist PhD student or to the research staff from the Center of Experimental Therapeutics (CHUV), who will present the informed consent form to the patient after the medical visit. The participants will follow their treatment as usual, and no changes in the usual medical procedure will be made. No payment or compensation will be provided to the patients or to the oncologists as the patients are being seen as part of their routine follow-up clinical care".<br><br>As this information is already in the published protocol available open-source, and as this article is already quite long, we did not add this information in the current paper.
2. Which theoretical framework guided the study design and the selection of variables?	The study design and protocol were both built by an interprofessional team of experts in medication adherence, statistics, and medical oncology. The selection of variables was decided after several iterative discussions until a consensus was reached, based on the expertise of the investigators and based on the scientific literature. We did not use a theoretical framework to design the study or to select the variables.
3. Could you please elaborate on the intervention process and the content that was implemented during the study?	The process of the intervention and its implementation were described in the methods section, from lines 220 to 246.
4. What was the effect size and power of the study?	The power calculation has been provided in the protocol that has been published open source: Bandiera et al. JMIR Res Protoc. 2021 Jun 29;10(6):e30090. doi:

	10.2196/30090. PMID: 34185020; PMCID: PMC8278299.
How did you calculate and make sure the study sample size was large enough to have sufficient power for statistical analyses? Please provide more information to address these issues.	The sample size calculation was presented in the published protocol[7], cited lines 195-196.<br>In the published protocol, the sample size calculation is described as follows: "We assumed that the mean longitudinal adherence will remain stable at 95% in the intervention group during the follow-up period, whereas it would decrease from 95% to 80% in the control group during the 340 days from randomization to the end of follow-up. We also assumed an SD for the logit of longitudinal adherence of 1.8, for both groups and at all timepoints. This corresponds to 95% individual adherence in the intervals 10%-99.3% and 35%-99.9% in the control and intervention groups, respectively, at the end of follow-up. A sample size calculation was performed to simulate individual series of daily medication (1=at least the correct number of daily openings of the electronic monitor; 0=fewer daily openings than prescribed) according to the above parameters to consider both the interindividual variability (SD 1.8) and the intraindividual variability (the measurement error). Considering that the number of measures of a subject will be 340 and assuming a 10% dropout in the middle of the year, 120 patients must be included in the study (60 in each group) to reach a power of 80% with a .05 significance level when comparing the mean of the logit of global adherence between groups using a two-tailed Student *t* test. Differences are considered statistically significant if *P*<.05. »<br><br>Finally, the risk of contamination of the control group has been extensively discussed in the limitations section, lines 603-623.

Results<br>1. Why did the two groups exhibit such a huge disparity in the median time spent on adherence after enrollment?	The disparity in the time spent in the intervention versus control has been discussed in the discussion section, lines 623-630: “The attrition rate was high in our study, which is aligned with the high attrition rates in oncology trials[8]. Indeed, numerous patients dropped out due to cancer progression, as expected and reported in the literature[9]. Other reasons for attrition were related to the burden of the study, which we aimed to alleviate”.<br><br>The reasons of the drop-outs in each group are presented in Figure 2, line 429. In both groups, the main reason for the premature stop of the study was the stop of the OAT; cancer progression was the first cause, followed by side effects.
Discussion<br>1. Regarding medication implementation, the intervention benefited mostly men, patients younger than 60 years, patients prescribed PKIs longer than 60 days, patients without a diagnosis of metastasis or with a metastatic disease experience longer than 2 years, and patients who had never used any adherence tool in their therapeutic itinerary.<br>→ What could be the potential explanations for the factors identified in relation to medication implementation?	The discussion of the results has been provided in the discussion section “3. Determinants of implementation”, lines 545-561. We hope this answers the question.
Do the study findings align with the existing literature?<br><br>You need to address these issues in the discussion section.	The study findings were compared to the literature in the discussion section, lines 545-561.
2. First, the OpTAT study explored adherence to PKIs among patients with advanced solid cancers,…<br>→ Was the cancer stage used as a criterion for participant recruitment? Without such criteria, making statements about advanced solid cancers may not be justified.	Inclusion and exclusion criteria are presented on lines 196-199. The cancer stage was not used as a criterium for participant eligibility. However, as seen in Table 1, most PKIs were prescribed as palliative lines (> 80%), and most of the patients were diagnosed with a metastasis, which indicate a cancer stage 4. We agree with the reviewer that PKIs have not always been prescribed in advanced solid cancer in our study. Thus, we clarified by removing the word “advanced”, line 584: “the OpTAT study

	explored adherence to PKIs among patients with solid cancers(…)”.

Reviewer #2:

The study is rigorous, clear and accurate, I would however reformulate the hypothesis.<br>The statements below are not clear and prone to confusion:<br>- We hypothesized that PKI implementation would be significantly lower if the alternate regimens (i.e., transient interruptions of PKI) prescribed by oncologists were not considered in the calculation<br>- Persistence would be improved<br>- These patients would perceive fewer prejudices and concerns about taking PKIs.	Thank you for your comment.<br>We have clarified the following hypothesis as follows: “We hypothesized that considering PKIs alternate regimens (i.e., transient interruptions of PKI) in the implementation calculation, rather than the usual regimens recommended by the pharmaceutical industry, would allow us to highlight the risk of underestimating the implementation outcome, when researchers do not consider the alternative regimens that often occur in routine care.” (lines 150-154).<br>We clarified the hypothesis regarding the persistence, line 144-145: “(…) that patients in the intervention group would persist longer on PKIs ».<br>We also clarified the hypothesis on patients’ beliefs and concerns, lines 146-147, “We further hypothesized that (…) the intervention would influence patients’ beliefs and reduce patients’ concerns about taking PKIs”.

Reviewer #3:

I have some concerns on the study design and statistical analysis.<br><br>1. The authors need to provide the sample size calculation and power analysis to justify the design of the study.	Thank you for your comment.<br>The sample size calculation was presented in the published protocol[7], cited line 195-196.<br>In the published protocol, the sample size calculation was described as follows: “We assumed that the mean longitudinal adherence will remain stable at 95% in the intervention group during the follow-up period, whereas it would decrease from 95% to 80% in the control group during the 340 days from randomization to the end of follow-up. We also assumed a SD for the logit of longitudinal adherence of 1.8, for both groups and at all timepoints. This corresponds to 95% individual adherence in the intervals 10%-99.3%

	and 35%-99.9% in the control and intervention groups, respectively, at the end of follow-up. A sample size calculation was performed to simulate individual series of daily medication (1=at least the correct number of daily openings of the electronic monitor; 0=fewer daily openings than prescribed) according to the above parameters to consider both the interindividual variability (SD 1.8) and the intraindividual variability (the measurement error). Considering that the number of measures of a subject will be 340 and assuming a 10% dropout in the middle of the year, 120 patients must be included in the study (60 in each group) to reach a power of 80% with a .05 significance level when comparing the mean of the logit of global adherence between groups using a two-tailed Student *t* test. Differences are considered statistically significant if *P*<.05. »[7]<br><br>As this article is already long, we have not repeated the sample size calculation in this paper, but we have cited the source so that readers can easily refer to it.
2. There are multiple testing that have been conducted in the study. However, there is no description on multiple comparison adjustment to avoid the inflation of false positive.	The reviewer refers to the fact that we estimated several models to see the effect of each covariate (gender, age, time since diagnosis, etc.) on PKI implementation. We are aware that this could generate multiple testing problems, and that it does not allow us to estimate the effect of each covariate adjusted for the value of the others. However, the complexity of the model, including, in addition to the risk factor, a polynomial effect of time, and an interaction between the risk factor and time (necessary if we want to estimate different time trajectories according to the value of the covariate and not just the difference at baseline) prevented us from estimating a multivariate model. For this reason, this part of the study should be considered more as an exploratory analysis to see how implementation might

	vary over time according to patient characteristics, than as a truly explanatory analysis in the sense of statistical significance. This information has been added to the manuscript on line 354: "Patient age, gender, use of adherence tools, time between PKI initiation and study inclusion, time between metastatic diagnosis and study inclusion, and the presence of distant metastases were included as covariables one at a time in the GEE model as an exploratory analysis."
3. In Figure 5, there is no methodology description on how to generate the curves of the empirical implementation.	Empirical implementation is defined lines 334-336: "On each day of the monitoring period, empirical implementation was defined by the proportion of patients with correct medication intake (proportion of outcomes=1) among patients still participating in this study that day[10]". In order to clarify how the curves were generated, we added line 336-338 "For example, implementation on day d is x/y=z%, since x out of y patients still under observation on day d were taking their medication according to their prescription."
4. The major comparison is between the intervention and control groups, suggest removing the 9 patients of the not randomized from Table 1.	Thank you for your comment.<br>The 9 patients that were not randomized did not benefit from the intervention, as they left the study during the baseline period. Their implementation outcomes contributed to implementation estimate during the first weeks of the baseline period. They are treated in the model as patients staying in control group for some weeks before being lost to follow-up (e.g., a patient was not randomized as he/she stayed 19 days in the study), in the same way as a patient randomized in the control around day 21 and lost to follow-up right after (e.g., a patient was randomized 20 days after inclusion and left the study 10 days after randomization). It is important to note that we have not two separate groups under the entire duration of the

	study, but rather two states, control and intervention, that can be visited at different times during the study by a same individual (time dependent variable). In this sense the 9 patients above are not different from the other as they are (constantly) in one of these two states before they are lost.<br>This information has been added as footnote[d] of Table 1 line 411-414.

Journal requirements

Please ensure that your manuscript meets PLOS ONE's style requirements, including those for file naming.	We renamed the files as required by PLOS ONE.
We note that you have indicated that data from this study are available upon request. PLOS only allows data to be available upon request if there are legal or ethical restrictions on sharing data publicly.<br><br>a) If there are ethical or legal restrictions on sharing a de-identified data set, please explain them in detail (e.g., data contain potentially identifying or sensitive patient information) and who has imposed them (e.g., an ethics committee).	Indeed, data of this study are sensible, e.g., medication implementation at each day for each patient. As these data are very specific for each patient, we cannot exclude that patients could be recognized through their implementation outcomes, especially for low implementation schemes. As the database contains potentially identifying data, the data are not provided in open access.
We notice that your supplementary figures and tables (Appendix 1-4) are included in the manuscript file. Please remove them and upload them with the file type 'Supporting Information'. Please ensure that each Supporting Information file has a legend listed in the manuscript after the references list.	We removed the Appendix 1-4 in the manuscript and we uploaded them as “Supporting Information”. We ensured that each supporting information file has a legend listed in the manuscript after the references list.
We note that the original protocol that you have uploaded as a Supporting Information file contains an institutional logo. As this logo is likely copyrighted, we ask that you please remove it from this file and upload an updated version upon resubmission.	The protocol of the OpTAT study has been published in JMIR Reseach Protocol in 2021[7] (Bandiera C, Cardoso E, Locatelli I, Digklia A, Zaman K, Diciolla A, Cristina V, Stravodimou A, Veronica AL, Dolcan A, Sarivalasis A, Liapi A, Bouchaab H, Orcurto A, Dotta-Celio J, Peters S, Decosterd L, Widmer N,

	Wagner D, Csajka C, Schneider MP. Optimizing Oral Targeted Anticancer Therapies Study for Patients With Solid Cancer: Protocol for a Randomized Controlled Medication Adherence Program Along With Systematic Collection and Modeling of Pharmacokinetic and Pharmacodynamic Data. JMIR Res Protoc. 2021 Jun 29;10(6):e30090. doi: 10.2196/30090. PMID: 34185020; PMCID: PMC8278299).<br><br>As the protocol is open source, we do not consider it necessary to upload it as a Supplementary File. Please let us know.<br><br>If the protocol of the OpTAT needs to be cited as part of this current publication, please cite the protocol published in JMIR with the citation provided above.
We note that the original protocol file you uploaded contains a confidentiality notice indicating that the protocol may not be shared publicly or be published. Please note, however, that the PLOS Editorial Policy requires that the original protocol be published alongside your manuscript in the event of acceptance. Please note that should your paper be accepted, all content including the protocol will be published under the Creative Commons Attribution (CC BY) 4.0 license, which means that it will be freely available online, and any third party is permitted to access, download, copy, distribute, and use these materials in any way, even commercially, with proper attribution.<br><br>Therefore, we ask that you please seek permission from the study sponsor or body imposing the restriction on sharing this document to publish this protocol under CC BY 4.0 if your work is accepted. We kindly ask that you upload a formal statement signed by an institutional representative	The OpTAT protocol has been published elsewhere [7] and is available in open access: Bandiera et al. JMIR Res Protoc. 2021 Jun 29;10(6):e30090. doi: 10.2196/30090. PMID: 34185020; PMCID: PMC8278299. We hope this meets the Editorial policy requirements of PLOS. We hope this answers your query.

clarifying whether you will be able to comply with this policy. Additionally, please upload a clean copy of the protocol with the confidentiality notice (and any copyrighted institutional logos or signatures) removed.	

We have uploaded the required documents in the submission portal.
Thank you for your consideration,
Sincerely,

Dr Carole Bandiera, Dr Isabella Locatelli and Prof. Marie P. Schneider

References

1. Kardas, P., P. Lewek, and M. Matyjaszczyk, *Determinants of patient adherence: a review of systematic reviews.* Front Pharmacol, 2013. **4**: p. 91.
2. Naser, A.Y., et al., *Real World Adherence to and Persistence With Oral Oncolytics in Multiple Myeloma: A Systematic Review and Meta-analysis.* Clin Lymphoma Myeloma Leuk, 2022. **22**(10): p. 760-773.
3. Atkinson, T.M., et al., *The Association Between Patient-Reported and Objective Oral Anticancer Medication Adherence Measures: A Systematic Review.* Oncol Nurs Forum, 2016. **43**(5): p. 576-82.
4. Greer, J.A., et al., *A Systematic Review of Adherence to Oral Antineoplastic Therapies.* Oncologist, 2016. **21**(3): p. 354-76.
5. Huang, W.C., et al., *Medication adherence to oral anticancer drugs: systematic review.* Expert Rev Anticancer Ther, 2016. **16**(4): p. 423-32.
6. Murphy, C.C., et al., *Adherence to adjuvant hormonal therapy among breast cancer survivors in clinical practice: a systematic review.* Breast Cancer Res Treat, 2012. **134**(2): p. 459-78.
7. Bandiera, C., et al., *Optimizing Oral Targeted Anticancer Therapies Study for Patients With Solid Cancer: Protocol for a Randomized Controlled Medication Adherence Program Along With Systematic Collection and Modeling of Pharmacokinetic and Pharmacodynamic Data.* JMIR Res Protoc, 2021. **10**(6): p. e30090.
8. Hui, D., et al., *Attrition rates, reasons, and predictive factors in supportive care and palliative oncology clinical trials.* Cancer, 2013. **119**(5): p. 1098-105.
9. Watson, G.A., et al., *Real-World Experience of Palbociclib-Induced Adverse Events and Compliance With Complete Blood Count Monitoring in Women With Hormone Receptor-Positive/HER2-Negative Metastatic Breast Cancer.* Clin Breast Cancer, 2019. **19**(1): p. e186-e194.
10. Schneider, M.P., et al., *A Novel Approach to Better Characterize Medication Adherence in Oral Anticancer Treatments.* Frontiers in Pharmacology, 2019. **9**(1567).
